# Wearable Devices to Diagnose and Monitor the Progression of COVID-19 Through Heart Rate Variability Measurement: Systematic Review and Meta-Analysis

**DOI:** 10.2196/47112

**Published:** 2023-11-14

**Authors:** Carlos Alberto Sanches, Graziella Alves Silva, Andre Felipe Henriques Librantz, Luciana Maria Malosa Sampaio, Peterson Adriano Belan

**Affiliations:** 1 Informatics and Knowledge Management Graduate Program Universidade Nove de Julho São Paulo Brazil

**Keywords:** heart rate variability, HRV, wearable device, COVID-19, SARS-CoV-2, wearable, diagnosis, mobile phone

## Abstract

**Background:**

Recent studies have linked low heart rate variability (HRV) with COVID-19, indicating that this parameter can be a marker of the onset of the disease and its severity and a predictor of mortality in infected people. Given the large number of wearable devices that capture physiological signals of the human body easily and noninvasively, several studies have used this equipment to measure the HRV of individuals and related these measures to COVID-19.

**Objective:**

The objective of this study was to assess the utility of HRV measurements obtained from wearable devices as predictive indicators of COVID-19, as well as the onset and worsening of symptoms in affected individuals.

**Methods:**

A systematic review was conducted searching the following databases up to the end of January 2023: Embase, PubMed, Web of Science, Scopus, and IEEE Xplore. Studies had to include (1) measures of HRV in patients with COVID-19 and (2) measurements involving the use of wearable devices. We also conducted a meta-analysis of these measures to reduce possible biases and increase the statistical power of the primary research.

**Results:**

The main finding was the association between low HRV and the onset and worsening of COVID-19 symptoms. In some cases, it was possible to predict the onset of COVID-19 before a positive clinical test. The meta-analysis of studies reported that a reduction in HRV parameters is associated with COVID-19. Individuals with COVID-19 presented a reduction in the SD of the normal-to-normal interbeat intervals and root mean square of the successive differences compared with healthy individuals. The decrease in the SD of the normal-to-normal interbeat intervals was 3.25 ms (95% CI −5.34 to −1.16 ms), and the decrease in the root mean square of the successive differences was 1.24 ms (95% CI −3.71 to 1.23 ms).

**Conclusions:**

Wearable devices that measure changes in HRV, such as smartwatches, rings, and bracelets, provide information that allows for the identification of COVID-19 during the presymptomatic period as well as its worsening through an indirect and noninvasive self-diagnosis.

## Introduction

### Background

COVID-19 was declared a pandemic by the World Health Organization in March 2020 because of the global logarithmic expansion of cases, which resulted in considerable morbidity and mortality. Although the respiratory system is predominantly affected, multiple organ dysfunctions, including cardiac injury, have been widely reported [1,2].

Heart rate variability (HRV) is a reliable marker of several physiological factors modulating the normal rhythm of the heart. HRV is a noninvasive, objective, and validated measurement of autonomic nervous system dysfunction, providing information on the balance between the sympathetic and parasympathetic nervous systems represented by variations in the time intervals between consecutive heartbeats [3]. Factors that affect HRV can be divided into different groups, including nonmodifiable, environmental, physiological, pathological, and lifestyle factors [4,5] (Textbox 1).

**Definition of the different factors that affect heart rate variability (HRV).**

**Nonmodifiable factors**

- With age, HRV decreases, and women appear to be more affected than men by this decline [6,7].

**Environmental factors**

- A systematic review listed several factors related to the work environment that can influence the reduction in HRV [8]. Among these factors are the electromagnetic field, vibrating tools, psychosocial workload, fatigue, working time, and 24-hour work shifts.

**Physiological factors**

- Age, sex, ethnicity, and circadian rhythm influence HRV [4,9].

**Pathological factors**

- In the study by Papaioannou et al [10], they concluded that HRV alterations during critical illness such as sepsis and multiple organ dysfunction syndrome demonstrate that loss of variability in heart rate signals is inversely correlated with immune response, particularly in most severe cases.

**Lifestyle factors**

- It has been observed that cigarette smoking has a negative effect on autonomic function with reduced HRV [11,12], whereas low doses of alcohol intake in nondependent users (1 standard drink in women and 2 in men daily) are linked to increased HRV and greater alcohol intake is linked to decreased HRV [13].

In addition to frequently used long-term HRV analysis (≥24 hours), short-term HRV analysis (approximately 5 minutes) is increasingly being applied because of near real-time test results [14,15], and the recent advances in this field suggest the use of ultra–short-term (<5 minutes) HRV measurements [16]. Ultrashort HRV has grown, especially in combination with mobile phones, smartwatches, and wearable sensors to monitor an individual’s state of health and well-being [17].

In general, although a high HRV is associated with good health, alternatively, a reduction in HRV is related to health problems [4,5]. The relationship between the different indexes of HRV and inflammatory markers has been widely studied, and a meta-analysis of 51 studies has demonstrated that HRV indexes are inversely related to the levels of the inflammatory markers [18]. Recent studies have associated reductions in HRV with COVID-19 [19-24].

Traditional methods of measuring HRV, including equipment such as electrocardiograms (ECGs), Holter monitors, and cardiac belts, are used in clinical settings. In contrast, wearable devices are noninvasive body-worn sensors that automatically monitor physiological signals [25] and are revolutionizing the way of identifying individual changes in these parameters [26,27] through mobile and digital health by enabling continuous health monitoring everywhere and anytime, from hospitals to in-home disease management [28,29]. Furthermore, the validity, reliability, and accuracy of commercial wearables in the measurement of different physiological parameters have improved in recent years [30,31].

Hernando et al [31] compared the HRV parameters extracted from an Apple Watch device with those extracted from a Polar H7 band during relaxation and mental stress in 20 healthy volunteers. No substantial differences were found when comparing temporal HRV indexes derived from the RR interval series provided by both devices. However, the low-frequency (LF) and high-frequency (HF) powers were substantially different when derived from the Apple Watch.

A recent study with 263 people using a smartwatch with a simultaneous recording-derived photoplethysmography (PPG) signal and a high-resolution (1000 Hz) ECG for 30 minutes under standard conditions demonstrated that HRV markers can be calculated from a smartwatch’s PPG signal at rest [32].

Cao et al [33] tested the Oura Ring device and observed that it was possible to accurately measure nocturnal heart rate (HR) and the root mean square of the successive differences (RMSSD) in both the 5-minute and average-per-night tests. In addition, the device provided acceptable accuracy in nocturnal average of normal-to-normal heartbeat intervals, percentage of successive normal beat-to-beat intervals that differ by >50 ms, HF, and SD of the normal-to-normal interbeat intervals (SDNN) in the average-per-night test but not in the 5-minute test. In contrast, the tests in the LF domain and LF:HF ratio had high error rates in both situations.

The systematic review conducted by Georgiou et al [34] revealed that commercial wearable devices, especially those using PPG, may provide a promising alternative solution for measuring HRV but that they can only be used as a surrogate in resting or mild exercise conditions as their accuracy diminishes with increasing exercise load.

The combination of these factors has led different researchers to evaluate the use of wearable medical devices to determine the onset of COVID-19 and its severity and, sometimes, predict the mortality of infected people through physiological signals [35-37].

### Objectives

The objective of this study was to assess the utility of HRV measurements obtained from wearable devices as predictive indicators of COVID-19, as well as the onset and worsening of symptoms in affected individuals.

## Methods

### Protocol and Registration

The protocol for this review was registered in PROSPERO (CRD42023399705). This review was conducted according to the PRISMA (Preferred Reporting Items for Systematic Reviews and Meta-Analyses) guidelines [38] (Multimedia Appendix 1 [38]).

### Eligibility Criteria

#### Inclusion Criteria

Studies that fulfilled the following criteria were included: (1) HRV measurements focused on COVID-19 and (2) measurements involving wearable devices.

#### Exclusion Criteria

The exclusion criteria comprised studies (1) in which the purpose of HRV analysis was unrelated to COVID-19; (2) that did not report numerical values for HRV variation or in which the measurements were taken using regular ECG equipment; (3) in which, even though they provided information about HRV, the analysis was performed on other physiological data related to COVID-19; (4) that were systematic reviews (autonomic dysfunction, performance of wearable sensors, and impact of SARS-CoV-2 infection on HRV); (5) that focused on long-term COVID-19; (6) that were conference abstracts; and (7) that did not mention the source of the information.

### Search Strategy

The following databases were searched: Embase, PubMed, Web of Science, Scopus, and IEEE Xplore. The search was designed to identify studies linking low HRV with COVID-19. The following terms were used for all databases searched: (COVID OR “COVID-19” OR “SARS-COV-2”) AND (“Heart Rate Variability” OR HRV). No language restrictions were applied, and the search period was all-inclusive up to the end of January 2023.

### Study Selection

The search returned 1112 records: 332 (29.86%) from Embase, 293 (26.35%) from Scopus, 256 (23.02%) from Web of Science, 202 (18.17%) from PubMed, and 25 (2.25%) from IEEE Xplore. In addition, 4 articles identified through manual searches were included.

The screening of the articles was conducted in a 2-step process. First, the first author (CAS) removed duplicates and screened the titles, abstracts, and conclusions of the reports. The second author (GAS) then verified the decisions made. Second, the texts of the remaining articles were read by the first author (CAS) to create a short list. The short-listed and removed articles were then verified by the second author (GAS). Any disagreements during the selection process were resolved by a third author (AFHL, LMMS, or PAB).

**Figure 1:**
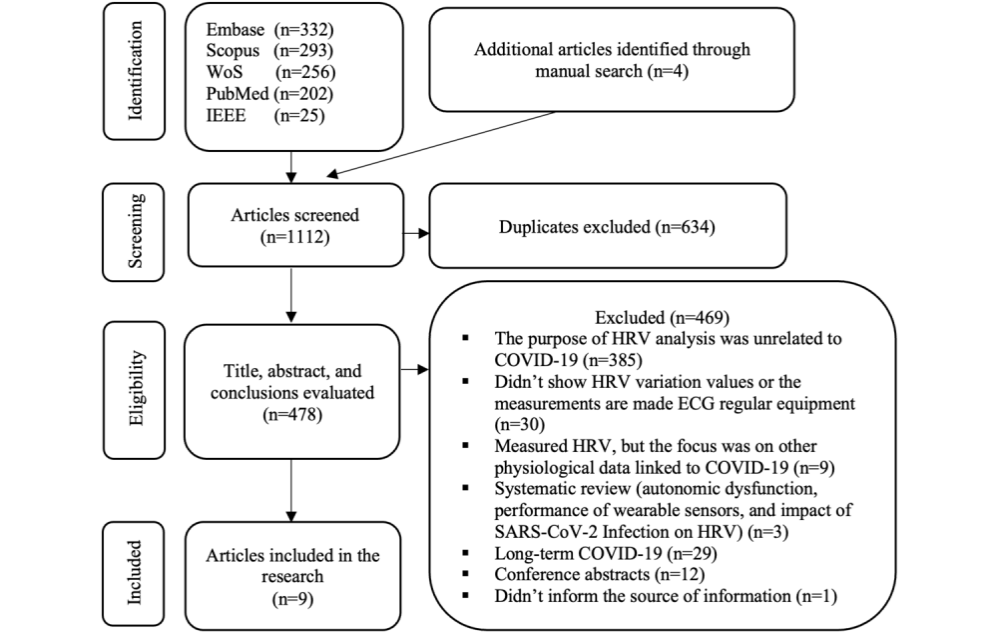
Flowchart of study selection.

After eliminating duplicates, 42.99% (478/1112) of the results were evaluated by title, abstract, and conclusions. Of these 478 studies, 385 (80.5%) did not link changes in HRV with COVID-19, a total of 30 (6.3%) did not show HRV variation values or took the measurements using standard ECG equipment, 9 (1.9%) focused on other physiological data linked to COVID-19 despite measuring HRV, 29 (6.1%) focused on long-term COVID-19, a total of 12 (2.5%) were conference abstracts, 3 (0.6%) were systematic reviews (autonomic dysfunction, performance of wearable sensors, and impact of SARS-CoV-2 infection on HRV), and 1 (0.2%) did not report the source of the data used. Therefore, these studies were excluded. As a result, 9 articles were included in the full-text review stage [39-47]. Details of the selection process are shown in Figure 1.

### Data Extraction

In total, 2 authors (CAS and GAS) independently retrieved the data from each study, included them in the final selection, and reported the variables of interest in a spreadsheet file. The extracted data included first author and year of publication, sample size and demographic characteristics of the study population, devices used for the measurement of HRV, main variation in HRV index, and whether the study attempted to predict SARS-CoV-2 infection before symptom onset or only analyzed its effect on HRV during the infection period. Any disagreements were resolved through discussion and reference to the other 3 authors (AFHL, LMMS, and PAB).

### Meta-Analysis

This analysis focused on HRV time-domain parameters, specifically the SDNN and RMSSD, both measured in milliseconds.

A total of 56% (5/9) of the studies [40-43,47] were subjected to a meta-analysis based on fixed effects performed using RevMan (version 5.4; The Cochrane Collaboration). In total, 22% (2/9) of the studies [39,45] could not be included in the meta-analysis as the results were presented in *Z* score and fractional values, whereas all the other studies presented values in milliseconds. Another 22% (2/9) of the studies [44,46] were not included because of the small number of participants involved (N=1 and N=2).

### Assessment of the Risk of Bias

All the identified articles and their methodological quality were independently evaluated by 2 authors (CAS and GAS), and a consensus was reached by consulting a third author (LMMS) if necessary. The methodological quality of observational studies (cohort and case-control) was determined using the tools of the Joanna Briggs Institute (JBI) [48].

## Results

### Type of Studies

Of the 9 included studies, all (100%) were observational, 4 (44%) were prospective cohort studies [41-43,47], another 3 (33%) were retrospective studies, 2 (22%) were cohort studies [39,40,45], and 2 (22%) were case studies [44,46].

### Population and Characteristics

Most studies (6/9, 66%) included a population with a mean age of <44 years, and only 33% (3/9) included a population with a mean age of >51 years [41,42,44]. The age of the participants ranged from 18 to 84 years, and female participants represented 76.78% (8129/10,588), with the proportion of female participants ranging from 29% to 100% across studies, and 11% (1/9) of the studies having only male participants [44]. The sample sizes ranged from 1 to 7200, with 33% (3/9) of the studies presenting data on participant ethnicity [41,43,46]. COVID-19 was primarily diagnosed using a real-time polymerase chain reaction (RT-PCR) test. In 22% (2/9) of the studies, the diagnosis of COVID-19 was self-reported by the participants [40,45], and in another study, both the RT-PCR and antibody tests were used [47]. Finally, 11% (1/9) of the studies did not specify the test used to diagnose infection [42]. Regarding severity, symptoms ranged from asymptomatic to critical, with most participants reporting mild symptoms. One study (1/9, 11%) was unclear about the severity of COVID-19, a total of 22% (2/9) did not report symptoms [40,47], and the COVID-19 variant was not reported in any of the studies.

### Risk of Bias

Applying the JBI Critical Appraisal Checklist for Cohort Studies [48] criteria, 56% (5/9) of the studies [39-42,45] were classified as having a high risk of bias, and 22% (2/9) [43,47] were classified as having a low risk of bias (Table 1).

**Table 1:** Risk of bias of cohort studies.

Study	Q1^a^	Q2^b^	Q3^c^	Q4^d^	Q5^e^	Q6^f^	Q7^g^	Q8^h^	Q9^i^	Q10^j^	Q11^k^	Overall
Natarajan et al [39], 2020	H^l^	L^m^	L	H	H	H	L	L	L	H	L	H
Hijazi et al [40], 2021	H	H	L	H	H	N/A^n^	L	L	L	H	L	H
Hasty et al [41], 2021	H	L	L	H	H	U^o^	L	L	L	H	L	H
Lonini et al [42], 2021	H	L	L	H	L	U	L	U	L	H	L	H
Hirten et al [43], 2021	L	L	L	H	L	U	L	U	L	H	L	L
Natarajan et al [45], 2022	H	L	L	H	L	U	L	U	L	H	L	H
Risch et al [47], 2022	L	L	L	H	L	U	L	U	L	H	L	L

^a^Q1: Were the 2 groups similar and recruited from the same population?

^b^Q2: Were the exposures measured similarly to assign people to both the exposed and unexposed groups?

^c^Q3: Was the exposure measured in a valid and reliable way?

^d^Q4: Were confounding factors identified?

^e^Q5: Were strategies to deal with confounding factors stated?

^f^Q6: Were the groups or participants free of the outcome at the start of the study (or at the time of exposure)?

^g^Q7: Were the outcomes measured in a valid and reliable way?

^h^Q8: Was the follow-up time reported and long enough for outcomes to occur?

^i^Q9: Was follow-up complete, and if not, were the reasons for loss to follow-up described and explored?

^j^Q10: Were strategies to address incomplete follow-up used?

^k^Q11: Was appropriate statistical analysis used?

^l^H: high.

^m^L: low.

^n^N/A: not applicable.

^o^U: unclear.

Applying the JBI Critical Appraisal Checklist for Cohort Studies [48] criteria, 11% (1/9) of the studies [44] were classified as having an unclear risk of bias, and another one [46] was classified as having a low risk of bias. Both studies were assessed on the following questions: Q1: Were patients’ demographic characteristics clearly described? Q2: Was the patients’ history clearly described and presented as a timeline? Q3: Was the current clinical condition of the patient on presentation clearly described? Q4: Were diagnostic tests or assessment methods and the results clearly described? Q5: Was the intervention or treatment procedure clearly described? Q6: Was the postintervention clinical condition clearly described? Q7: Were adverse events (harms) or unanticipated events identified and described? Q8: Does the case report provide takeaway lessons?

Gutiérrez et al [44] was classified as low risk of bias for questions Q1, Q2b, Q3, and Q4. However, the risk of bias was high for question Q6 and unclear for questions Q5, Q7, and Q8. Overall, the risk of bias in this study is unclear.

### Wearable Devices and Applied Technology

The bracelet design was the most commonly used, present in 56% (5/9) of the studies. Other studies included various devices, such as an armband, a chest strap, and an intelligent ring, and another study used a mechanoacoustic device. Most participants (10,144/10,588, 95.8%) measured their physiological data using the Fitbit device. PPG was used in 78% (7/9) of the studies [39-41,43,45-47]. PPG uses an optical sensor with a light source to measure the cyclical oscillations in the skin’s blood flow by emitting light to the skin and absorbing light reflection through a photosensitive diode. Variations in light intensity occur with a change in the volume of blood within the tissue with each heartbeat, providing information to the sensor [49,50]. One study used a chest strap [44], which, through electrodes positioned on the skin, detected changes in the electrical current in the heart in each heartbeat. Finally, one study used a mechanoacoustic sensor [42]. These sensors are highly responsive to movements and vibratory processes of the body, and their use at the suprasternal notch enables the acquisition of information related to several classes of physiological processes in the human organism [51].

### Physiological Data

The metrics used to assess HRV can be divided into 2 categories: time domain and frequency domain. The most common parameters in the time-domain evaluation include SDNN, percentage of successive RR intervals that differ by >50 ms, and RMSSD. In turn, frequency-domain measurements estimate the absolute or relative power distribution in 4 frequency bands: ultra-LF, very LF, LF, and HF [52]. All the evaluated studies obtained data related to HRV; however, 56% (5/9) also included other parameters, such as respiratory rate [39,45-47], walking cadence, and cough frequency spectrum [42]. The most common metrics used in the studies to assess HRV were the RMSSD [39,40,44-47] and SDNN [40-44,47], and 22% (2/9) of the studies also presented an analysis in the frequency domain [40,44]. Finally, 78% (7/9) of the studies also analyzed HR or resting HR [39,40,42,44-47] in addition to HRV. The summary of the results are provided in Table 2.

**Table 2:** Summary of the 9 studies that correlate changes in heart rate variability (HRV) indexes with COVID-19 using wearable devices.

Study	Study population	Demographic characteristics	Wearable device and applied technology	COVID-19 diagnostic test	Interest and comparison of studies
Natarajan et al [39], 2020	2745 COVID-19 positive	Mean age 41.2 (SD 12.8) years; 76% female	Fitbit; PPG^a^	RT-PCR^b^	HRV changes before and after RT-PCR test
Hijazi et al [40], 2021	186 COVID-19 positive	Mean age 40.2 (SD 17.2) years; 64% female	Fitbit, Garmin, and Apple watches; PPG	Self-reported by study participants	Tried to identify infection before SO^c^
Hasty et al [41], 2021	16 COVID-19 positive	Mean age 60.5 (SD 13.4) years; 71% male	Warfighter Monitor; ECG^d^	RT-PCR	HRV variation analysis during infection
Lonini et al [42], 2021	14 COVID-19 positive	Mean age 52 (SD 15.2) years; 50% female	Device developed by Northwestern University; mechanoacoustic	NR^e^	HRV variation analysis during infection
Hirten et al [43], 2021	297; 13/297 COVID-19 positive	Mean age 36.3 (SD 9.8) years; 69.4% female	Apple Watch; PPG	RT-PCR	Tried to predict infection before SO
Gutiérrez et al [44], 2022	1 COVID-19 positive	Aged 52 years; male	Polar H7; electrode (chest strap) ECG	RT-PCR	HRV variation analysis during infection
Natarajan et al [45], 2022	7200 COVID-19 positive	Mean age 41.7 (SD 13.3) years; 78.2% female	Fitbit; PPG	Self-reported by study participant	RMSSD^f^ changes before and after SO
Jimah et al [46], 2022	2 COVID-19 positive	Aged 24/25 years; 100% female; pregnant	Oura* *Ring; PPG	RT-PCR	RMSSD variation during infection
Risch et al [47], 2022	66 COVID-19 positive	Mean age 42.9 (SD 5.6) years; 72% female	Ava bracelet; PPG	72% RT-PCR; 28% antibody test	Tried to predict infection before SO

^a^PPG: photoplethysmography.

^b^RT-PCR: real-time polymerase chain reaction.

^c^SO: symptom onset.

^d^ECG: electrocardiogram.

^e^NR: not reported.

^f^RMSSD: root mean square of the successive differences.

### Outcomes

A total of 11% (1/9) of the studies aimed to investigate the feasibility of using physiological signals such as HRV to detect COVID-19 before the onset of symptoms [40], and another study focused on HRV changes as a possible predictive marker for the acute inflammatory response in patients with COVID-19, correlating the reduction in SDNN with worsening disease states [41].

Lonini et al [42] proposed a new paradigm based on the recording of responses of some physiological parameters, such as respiration rate, HR, and HRV, after a short sequence of physical activities and based on the variations in these parameters to indicate the presence of COVID-19.

Gutiérrez et al [44] followed the clinical evolution of a patient with COVID-19, monitoring HRV indexes in the time and frequency domains and identifying a reduction in the SDNN and RMSSD indexes during infection when compared with the recovery period, whereas another 33% (3/9) of the studies showed that, during the time of sickness, the RMSSD decreased, and this metric may change a few days before the onset of symptoms [39,45,46].

Finally, 22% (2/9) of the studies sought to predict infection before symptom onset. Hirten et al [43] collected data on HRV daily from health care workers at 1 hospital using a smartwatch and a custom app installed on their smartphones, seeking to predict infection before the appearance of any symptoms. Risch et al [47] revealed changes in different physiological parameters, including HRV, during incubation and the presymptomatic, symptomatic, and recovery periods of COVID-19 when compared with baseline and sought to predict infection before symptom onset based on these conditions. Table 3 summarizes the main findings and outcomes of each study.

**Table 3:** Summary of main changes in the heart rate variability (HRV) indexes in each study, including the main findings and end points.

Study	Main changes in the HRV indexes	Main findings and end points	Comparison
Natarajan et al [39], 2020	Observed a decrease in HRV after a positive test.	Relevant and predictive physiological signs related to COVID-19 may be detected by consumer wearable devices.	HRV was compared after a positive RT-PCR^a^ test vs before.
Hijazi et al [40], 2021	SDNN^b^, RMSSD^c^, and pNN50^d^ decreased from 2 days before SO^e^ until the end of the infection.	Presents a framework that uses physiological signals obtained from wearable devices in the presymptomatic screening of COVID-19 using different AI^f^ techniques to predict the disease before the onset of the symptoms.	HRV was compared after SO vs before in the same individuals.
Hasty et al [41], 2021	SDNN reduction correlates with worsening COVID-19 symptoms.	Daily HRV measurements can help in the triage, disease progression monitoring, and treatment of infection.	HRV was monitored in patients during the infection period.
Lonini et al [42], 2021	There was a lower SDNN value in infected patients when compared with the healthy group.	Wearable sensors can capture an array of cardiorespiratory parameters, which can help uncover physiological changes induced by respiratory diseases such as COVID-19.	HRV was compared in patients who were infected vs healthy controls after a positive RT-PCR test.
Hirten et al [43], 2021	Reductions in SDNN were observed from 7 days before to 7 days after a positive RT-PCR test.	HRV metrics collected from a common wearable device can identify SARS-CoV-2 infection during the presymptomatic period, in asymptomatic carriers, and before diagnosis through a PCR^g^ test.	HRV was compared after a positive RT-PCR test vs before.
Gutiérrez et al [44], 2022	RMSSD and SDNN decreased during the infection period.	Wearable devices at a low cost can be used to monitor the clinical evaluation of patients with COVID-19.	HRV was compared in the infection vs recovery period.
Natarajan et al [45], 2022	There was a lower RMSSD value in infected patients when compared with the healthy group.	Physiological metrics collected by common wearables devices show the reduction in RMSSD before COVID-19 SO.	HRV was compared in patients who were infected vs healthy controls after an RT-PCR test.
Jimah et al [46], 2022	RMSSD reduction was observed during SARS-CoV-2 infection.	Resting HRV is an important and sensitive indicator of COVID-19 during pregnancy.	HRV was compared after a positive RT-PCR test vs before.
Risch et al [47], 2022	SDNN was significantly reduced in the incubation, presymptomatic, and symptomatic periods of the disease.	The results suggest that the use of an ML^h^ algorithm together with a wearable device can serve as a promising tool for the presymptomatic or asymptomatic detection of COVID-19 before diagnosis through an RT-PCR test.	HRV was compared after a positive RT-PCR test vs before.

^a^RT-PCR: real-time polymerase chain reaction.

^b^SDNN: SD of the normal-to-normal interbeat intervals.

^c^RMSSD: root mean square of the successive differences.

^d^pNN50: the proportion of successive normal-to-normal intervals that differ by >50 ms.

^e^SO: symptom onset.

^f^AI: artificial intelligence.

^g^PCR: polymerase chain reaction.

^h^ML: machine learning.

### Meta-Analysis

**Figure 2:**
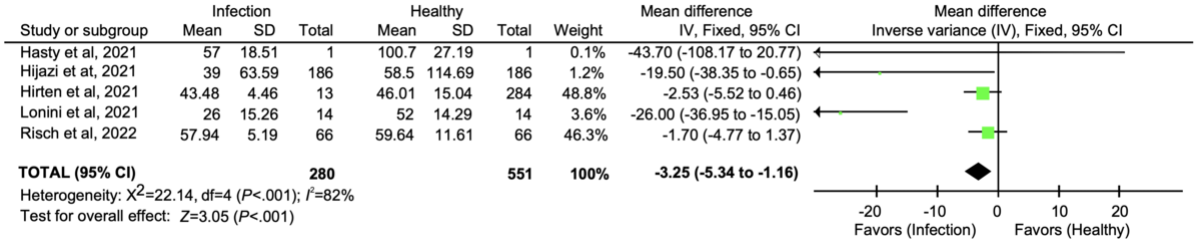
Results of SD of normal-to-normal interbeat interval variation.

**Figure 3:**
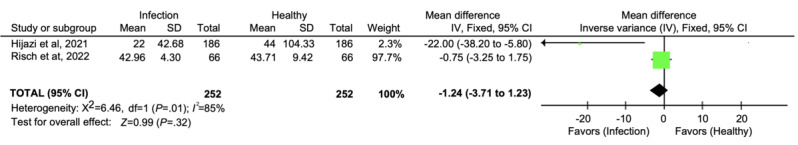
Results of root mean square of successive difference variation.

In total, 56% (5/9) of all studies were included in the meta-analysis. They were divided into 2 groups, one that presents the results of the SDNN index, which includes all studies (5/5, 100%; Figure 2 [40-43,47]), and another that presents the results of the RMSSD index, which includes 40% (2/5) of the studies (Figure 3 [40,47]).

Figure 2 shows the meta-analysis of all the studies that presented results of SDNN parameters, whereas Figure 3 shows the meta-analysis of studies that presented results of RMSSD.

These studies indicate that the HRV indexes are highly individualized, and this could explain the heterogeneity in the analysis of SDNN and RMSSD (*I*^2^=82% for SDNN and *I*^2^=85% for RMSSD in Figures 2 and 3, respectively).

Studies that created a baseline HRV for participants before SARS-CoV-2 infection [43,47] showed less variation in HRV values than studies that did not use this baseline [40-42].

The meta-analysis indicates that the reduction in the SDNN index was 3.25 ms (95% CI −5.34 to −1.16 ms) when comparing the healthy and infection periods (Figure 2) and the RMSSD index reduction was 1.24 ms (95% CI −3.71 to 1.23 ms) under the same conditions (Figure 3).

## Discussion

### Principal Findings

Our work suggests that devices used to assess HRV during SARS-CoV-2 infection may be reliable as the analyzed studies indicate that COVID-19–induced inflammation could affect the parasympathetic nervous system.

Although the meta-analysis statistically supported the idea that physiological measurements from wearable devices help detect and predict COVID-19 as well as its evolution through HRV measurement, there are some limitations in the quantification of the reduction in HRV indexes that can be associated with COVID-19. Another limitation is that our study only considered variations in the time domain, specifically the SDNN and RMSSD.

It is important to note that the pooled analyses of both the SDNN and RMSSD indexes showed high heterogeneity. Our subgroup analysis revealed that there was a quantitative difference in HRV variation (SDNN and RMSSD) between studies that previously established a baseline HRV in participants and studies that did not.

Sex and age have an influence on the HRV index for time- and frequency-domain measurements [53]. In the study by Bonnemeier et al [54], they investigated 166 healthy volunteers (81 women and 85 men aged 20-70 years) without evidence of cardiac disease and found significant differences in the time domain of HRV between female and male subgroups and a difference in measurements linked to age in both subgroups. Similarly, Sammito and Böckelmann [55] examined a group of 695 healthy participants of different ages and sexes with long-term 24-hour ECGs and observed a consistent decrease in HRV measurements with increasing age as well as a sex dependency of HRV values. Almeida-Santos et al [6] conducted a study with 1743 participants aged 40 to 100 years of both male and female sexes, functionally active, and with satisfactory cognitive function and found that the SDNN index decreased linearly with age and BMI and women had lower values than men. Koenig and Thayer [56] performed a meta-analysis that included 172 studies with reported data from 63,612 participants (31,970 female and 31,642 male) and found that women showed a lower mean RR interval and SDNN compared with men.

Hijazi et al [40] conducted a retrospective cohort study using a public data set, COVID-19 and Wearables Open Data Research [57], which comprises 186 participants. All of them were COVID-19 infected. Nonetheless, HRV measurements and readings before and after infection were available for some patients. Together with HRV measurements, this data set contained user textual logs reporting COVID-19–related symptom issues.

The authors sought to analyze the ability of artificial intelligence models to discriminate between healthy physiological signals (HRV) and affected physiological signals because of COVID-19. For this, they analyzed daily HRV changes, in addition to textual records from participants, as primary sources of information for different classification models to support the final decision. They found a mean reduction in SDNN between infection and healthy periods of 19.5 ms (95% CI −38.28 to −0.72 ms) and a decrease in RMSSD of 22.0 ms (95% CI −38.20 to −5.8 ms).

However, this data set presents high diversity in age, BMI, and measurement dates and collection times. Baseline values of physiological signs can be very different between participants, such as the HRV index of a woman aged 34 to 45 years with a BMI of 17.2 kg/m^2^ who lives in Russia and takes measurements in temperatures below 0 °C and that of a male individual aged 24 to 35 years with a BMI of 52.5 kg/m^2^ who lives in Qatar and takes measurements in temperatures close to 40 °C. In addition, the data collection process depended on the participants, who self-reported the onset and severity of symptoms, and physiological data were collected at different times of the day without information on the type of activity performed before this collection.

All these factors contribute to the heterogeneity of the data, making it difficult to establish a baseline of the HRV variation that can be used broadly to compare healthy and infected individuals. Using the same data set but restricted to 14 individuals with at least 5 high-quality measurements before and during SARS-CoV-2 infection, Ponomarev et al [58] found no differences in HRV values before, during, and after SARS-CoV-2 infection for group analysis. However, at the individual level, HRV showed statistically significant individual changes in some participants during SARS-CoV-2 infection.

Hasty et al [41], in a prospective study, analyzed data from 16 patients with positive polymerase chain reaction (PCR) test results for SARS-CoV-2 recruited in the intensive care unit. They took measurements of HRV and C-reactive protein (CRP) for each patient over a minimum of 7 days, with a data collection window of 5- to 7-minute intervals. Of these 16 patients, 12 developed a >50% increase in CRP level during the study period. Of the 12 patients who developed a >50% increase in CRP, 10 demonstrated a >40% drop in HRV within a 72-hour window preceding the increase in CRP.

In this study, the patient demographics and characteristics showed that 71% (12/17) had cardiovascular disease, 35% (6/17) had renal disease, 29% (5/17) had diabetes, and 24% (4/17) had pulmonary disease. All of them were admitted to the COVID-19 intensive care and step-down units, and this may represent a specific population condition as there were different comorbidities among the participants, with all presenting severe symptoms.

The numerical data of the SDNN variation were presented for only 1 patient, and this information was used in the meta-analysis. The reduction in SDNN was −43.7 ms (95% CI −108.17 to 20.77 ms). The consideration of SDNN values during the infection of this single patient may bias the analysis, in addition to the fact that they were all hospitalized and presenting with various comorbidities, restricting comparisons with other situations.

Gutiérrez et al [44] conducted a case study on a single patient, a man aged 52 years with moderate asthma, hypertension, and obesity who developed COVID-19 with moderate symptoms and no hospitalization. They obtained measurements during the second week of the patient’s illness; HRV was recorded from days 8 to 12, when symptoms worsened; and the last record was on the 19^th^ day, when the patient had almost recovered. The patient had a lower SDNN and RMSSD when ill than when he had recovered (SDNN: −53 ms, 95% CI −100.68 to −5.32 ms; RMSSD: −32.7 ms, 95% CI −62.40 to −3.07 ms).

These results show a high HRV variation comparing the period of SARS-CoV-2 infection with a period after the illness. However, there are at least 3 caveats to be considered before using these results. The first is the analysis of data from a single individual who had his own HRV baseline, which is affected by parameters such as age, sex, and BMI. The second is the presence of some comorbidities, especially asthma, which can influence the HRV results [59], and the third is the HRV data collection window with ultrashort periods of 2-minute intervals. In ultrashort HRV measurements (<5 minutes), a single misidentified heartbeat can alter HRV metrics [60]. This study was not included in the meta-analysis because of the number of participants (N=1).

Lonini et al [42] conducted a prospective cohort study recruiting 14 patients who tested positive for COVID-19 and required physical rehabilitation in 1 hospital, in addition to another 5 individuals who were recovering from the infection by quarantining at home and 14 healthy people. Of the 19 enrolled individuals infected with COVID-19, only 14 had usable data. Thus, the authors evaluated the physiological data of these 14 participants before and after a short sequence of activities (approximately 2 minutes) and compared them with those of a healthy group of 14 people.

The results showed that there was no intragroup difference in SDNN variation before the activities but there was a difference after the activities, and SDNN index variation was lower in the infected group. The reduction was 26.0 ms (95% CI −36.95 to −15.05 ms) compared with that of the healthy group.

The researchers captured an array of cardiorespiratory parameters during a short sequence of activities to monitor the HRV indexes. However, demographic differences between the 2 groups may bias the analysis of their findings. In this study, the control group had a lower mean age than the COVID-19–positive group (healthy controls: 32.4, SD 6.8 years; COVID-19 positive: 52.5, SD 15.7 years). In addition, BMI was lower in the healthy group (24.7, SD 2.6 kg/m^2^) than in the COVID-19–positive group (27.7, SD 7.5 kg/m^2^), and this can promote deviation in the outcomes. The differences in HRV measurements in demographically distinct groups are in line with the study by Almeida-Santos et al [6], who evaluated the HRV and patterns of autonomic regulation of the heart in 1743 individuals aged between 40 and 100 years who were functionally independent and had satisfactory cognitive function. Finally, the results presented are based on a specific type of sensor and the activities proposed by the researchers.

Hirten et al [43] conducted a prospective observational cohort study with 297 health care workers at a hospital with the primary objective of determining whether changes in the HRV index could differentiate healthy participants from those infected with COVID-19 as well as assessing whether these changes could predict the development of COVID-19 before a diagnosis through PCR testing. They found that the mean amplitude of the SDNN was different in patients with COVID-19 when compared with healthy participants and that the midline estimating statistic of rhythm was lower on the first day of symptoms compared with all other days.

They also reported that changes in HRV indexes were similar in patients with symptomatic and asymptomatic infections. The amplitude of the SDNN circadian rhythm between uninfected participants was 5.31 ms (95% CI 4.95-5.67 ms), whereas in infected participants, the amplitude 7 days before the COVID-19 diagnosis was 0.29 ms (95% CI –4.68 to 1.73 ms) and changed to 1.22 ms (95% CI –2.60 to 3.25 ms) 7 days after a diagnosis of COVID-19. The mean midline estimating statistic of rhythm on the first day of symptoms was 46.01 ms (95% CI 43.37-48.77 ms), and on all other days, it was 43.48 ms (95% CI 41.77-45.27 ms), and we used these values to conduct the meta-analysis.

The strengths of this study are the confirmation of COVID-19 through a positive PCR test and the demographic data showing a greater balance between the participants in average age (36.3, SD 9.8 years) and BMI (25.6, SD 5.7 kg/m^2^). In addition, all participants were health professionals working at a single hospital, which allowed for an average follow-up of 42 days, with a mean of 28 HRV samples per participant, allowing for more reliable data extraction. The main limitation was the small number of participants diagnosed with COVID-19 during data collection, which may have limited some analyses.

Jimah et al [46] conducted a continuous follow-up of 2 pregnant women during their SARS-CoV-2 infection in a case study. Both were tested using RT-PCR to confirm the infection and used the Oura Ring device to measure physiological signs. Between the third and sixth days, peak physiological changes in resting HR and HRV were observed. Between the third and sixth days after the onset of COVID-19 symptoms, both women experienced peak physiological changes in resting heart rate (HR) and heart rate variability (HRV). In both cases, there was a reduction in the RMSSD of 16.67 ms (95% CI −26.82 to −2.52 ms), which increased again after recovery.

The authors concluded that the HR, HRV, respiratory rate, and resting sleep stages are important and sensitive indicators of COVID-19 during pregnancy. However, physiological variations characterize pregnancy, and the autonomic nervous system is a critical regulatory system for the adaptations induced by pregnancy, which affected the HRV data in this study [61]. In addition, both participants had a prepregnancy BMI above the normal range, which may have also influenced the observed effects. This study was not included in the meta-analysis because of the number of participants (N=2).

Risch et al [47], through a prospective cohort study, investigated the possibility of using machine learning to try to predict infection before the onset of COVID-19 symptoms based on the detection of physiological changes through a wearable device. They collected physiological information from 66 participants using a wearable device to form a baseline (over 28 days) and analyzed changes in different physiological parameters across 4 periods related to COVID-19: incubation, presymptomatic, symptomatic, and recovery.

The device was used only while asleep and recorded the data every 10 seconds. In addition to the device, participants used a smartphone app that had custom functionality developed specifically for the COVID-19 study, and they recorded behaviors such as alcohol, medication, and drug intake in this app.

Compared with baseline, participants had a significant decrease in SDNN during the presymptomatic and postsymptomatic incubation periods. However, there was no statistically significant difference in RMSSD for participants who tested positive for COVID-19 during infection compared with the baseline, which differs from the results of other studies. The reduction in the SDNN baseline compared with the presymptomatic COVID-19 baseline was 1.70 ms (95% CI −4.77 to 1.37 ms), and for RMSSD, this reduction was 0.75 ms (95% CI −3.25 to 1.75 ms).

There were no statistically significant differences in sex ratio, age, or BMI between individuals who did or did not test positive for COVID-19 during follow-up. The combination of measurement time to create a baseline before infection, with COVID-19 confirmed via PCR (48/66) and antibody (18/66) tests, is a strength of this study. However, the small number of positive cases of COVID-19, lack of ethnic diversity, and age group without diversity (mean age 42.88, SD 5.59 years) may represent a bias in the observed results.

In total, 22% (2/9) of retrospective cohort studies [39,45] were not used in the meta-analysis as the numerical results were presented as *Z* scores and percentage changes. Nevertheless, the findings of these studies agree with those presented previously. In the study by Natarajan et al [39], they used physiological data from 2745 individuals diagnosed with COVID-19 (active infection; PCR test) residing in the United States and Canada collected through consumer wearable devices to train a neural network with the aim of predicting whether an individual was sick at a given time of the day considering certain physiological data such as the RMSSD and obtained an area under the curve of 0.77 (SD 0.018) for the prediction of infection on a specific day. In another study by Natarajan et al [45], they obtained physiological data from 7200 participants who reported a positive test for COVID-19 and from 1000 healthy participants, both through a consumer wearable device, and found that the mean RMSSD parameters of infected participants were different between men and women, with a reduction in RMSSD of 13.5% for men and 9.5% for women when comparing infected versus healthy participants. In this work, 79% (5688/7200) of the infected participants were women, and the age group between 20 and 49 years represented >70% of the participants, whereas in the control group, the individuals had a mean age of 45.3 (SD 13.9) years, and 71.6% (716/1000) of the participants were female.

Finally, a study that was not present in the meta-analysis because of having conducted the measurements using regular ECG equipment and, therefore, being within the exclusion criteria presented findings in opposition to all the aforementioned studies. Kaliyaperumal et al [62] compared some HRV parameters in 63 patients with COVID-19 with those of 43 healthy controls through an ambulatory 5-minute ECG and found that RMSSD was higher in the COVID-19 group than in healthy individuals (*P*=.02). In this study, the authors informed that both groups (healthy controls and infected participants) were instructed to abstain from smoking, consuming caffeine for 2 hours, and consuming alcohol for 36 hours and that they should have adequate rest, at least 8 hours of uninterrupted sleep, on the night before the HRV assessment, with a normal breakfast on the day of the assessment. This suggests that a single-point measurement was performed on each participant. The comparison of a single measurement between infected participants and healthy controls may not correctly represent the overall health status of the participants and is in contrast to the other studies reported in this paper.

### Limitations

This study has several limitations that should be considered. The meta-analysis was conducted using only time-domain HRV indexes, specifically SDNN and RMSSD, because of the limited availability of studies presenting HRV values in the frequency domain. Although these time-domain parameters are valuable indicators of HRV, they offer only a partial representation of HRV complexity.

The included studies exhibited heterogeneity in terms of participant number, study methodologies, and outcome measures. Although the overall findings showed similarities, quantitative differences were also observed. The impact of population size on the results remains unclear, and variations in the methodology applied may have contributed to variations in the results.

Furthermore, this review did not distinguish between different variants or subtypes of COVID-19 affecting the study participants. Variability in symptom severity and clinical manifestations among different participants could have influenced HRV patterns, and this heterogeneity was not accounted for in our analysis. It is important to note that other conditions characterized by systemic inflammation, such as sepsis or other viral infections, could also potentially affect HRV indexes.

### Conclusions

The increasingly extensive recording of individual characteristics and physiological data through wearable commercial devices will generate personal big data and require new strategies to develop individualized and data-driven health concepts.

This study is the first systematic review and meta-analysis to synthesize the existing literature that has assessed the diagnosis and monitoring of the progression of COVID-19 through the measurement of HRV using wearable devices.

The meta-analysis indicates that the overall results of HRV variation (SDNN and RMSSD) suggest that the reduction in these indexes can indicate an infection condition; the complexity lies in quantifying the range of values of decline in the HRV indexes (SDNN and RMSSD) that would characterize COVID-19. The studies that analyzed the average baseline of both infected and healthy populations exhibited less reductions in HRV indexes compared with studies that examined the individual baseline of participants during infection and healthy periods.

As several studies [6,53-56] have shown that HRV is individualized, the analysis of the variation in these indexes to diagnose and monitor the progression of COVID-19 should also consider this factor, and therefore, studies with a larger population are necessary but with individualized analyses.

Wearable devices that measure changes in HRV, such as smartwatches, rings, and bracelets, have shown potential in providing valuable information on COVID-19 during the presymptomatic period and its worsening through indirect and noninvasive self-diagnosis.

## Appendix

Multimedia Appendix 1 PRISMA (Preferred Reporting Items for Systematic Reviews and Meta-Analyses) checklist.

Multimedia Appendix 2 Summary of the data used in the meta-analysis.

## Abbreviations

CRP: C-reactive protein
ECG: electrocardiogram
HF: high frequency
HR: heart rate
HRV: heart rate variability
JBI: Joanna Briggs Institute
LF: low frequency
PCR: polymerase chain reaction
PPG: photoplethysmography
PRISMA: Preferred Reporting Items for Systematic Reviews and Meta-Analyses
RMSSD: root mean square of the successive differences
RT-PCR: real-time polymerase chain reaction
SDNN: SD of the normal-to-normal interbeat intervals

## Data Availability

This systematic review is based on an analysis of existing data from published studies. The data are publicly available from the original sources, which are cited in the References section. A summary of the data used in the meta-analysis is provided in Multimedia Appendix 2 [40-43,47].
