# Supplementary material for: Wearable Devices to Diagnose and Monitor the Progression of COVID-19 Through Heart Rate Variability Measurement: Systematic Review and Meta-Analysis
Source: J Med Internet Res. 2023 Nov 14;25:e47112. doi: 10.2196/47112 (PMC10685286; doi:10.2196/47112)
Supplement: Multimedia Appendix 2 [file jmir_v25i1e47112_app2.pdf]

Appendix 2 - Summary of the data used in the meta-analysis

Table 1 - Values used in the Meta-Analysis for SDNN

Author	Values	Location where the values was extracted from the article.
Hijazi et al, 2021 [40]	Infection 39 ms SD 63.59<br>Healthy 58.5 ms SD 114.69	Figure 8, page 12
Hasty et at, 2021 [41]	Infection 57 ms SD 18.51<br>Healthy 100.7 ms SD 27.19	Figure 2, page e36
Lonini et al, 2021 [42]	Infection 26 ms SD 15.26<br>Healthy 52 ms SD 14.29	Figure 2B
Hirten et. al, 2021 [43]	Infection 43.48 ms SD 4.46<br>Healthy 46.01 ms SD 15.04	Figure 3, page 14
Risch et al, 2022 [47]	Infection 57.94 ms SD 5.19<br>Healthy 59.64 ms SD 11.61	Table 3, page 9

Table 2 - Values used in the Meta-Analysis for RMSSD

Author	Values	Location where the values was extracted from the article.
Hijazi et al, 2021 [40]	Infection 22 ms SD 42.68<br>Healthy 44 ms SD 104.33	Figure 7, page 12
Risch et al, 2022 [47]	Infection 42.96 ms SD 4.3<br>Healthy 43.71 ms SD 9.42	Table 3, page 9
